# Azithromycin reduces inflammation in a rat model of acute conjunctivitis

**Published:** 2013-01-28

**Authors:** Patricia Fernandez-Robredo, Sergio Recalde, Maite Moreno-Orduña, Laura García-García, Javier Zarranz-Ventura, Alfredo García-Layana

**Affiliations:** 1 Experimental Ophthalmology Laboratory, Universidad de Navarra, Pamplona Navarra, Spain; 2 Ophthalmology, Clínica Universidad de Navarra, Pamplona, Navarra, Spain

## Abstract

**Purpose:**

Macrolide antibiotics are known to have various anti-inflammatory effects in addition to their antimicrobial activity, but the mechanisms are still unclear. The effect of azithromycin on inflammatory molecules in the lipopolysaccharide-induced rat conjunctivitis model was investigated.

**Methods:**

Twenty-four Wistar rats were divided into two groups receiving topical ocular azithromycin (15 mg/g) or vehicle. In total, six doses (25 µl) were administered as one dose twice a day for three days before subconjunctival lipopolysaccharide injection (3 mg/ml). Before the rats were euthanized, mucus secretion, conjunctival and palpebral edema and redness were evaluated. Real-time polymerase chain reaction was used to determine gene expression for interleukin-6, cyclooxygenase-2, tumor necrosis factor-α, matrix metalloproteinase (MMP)-2, and MMP-9. Interleukin-6 was determined with enzyme-linked immunosorbent assay, nuclear factor-kappa B with western blot, and MMP-2 activity with gelatin zymogram. Four eyes per group were processed for histology and subsequent periodic acid-Schiff staining and CD68 for immunofluorescence. The Student *t* test or the Wilcoxon test for independent samples was applied (SPSS v.15.0).

**Results:**

Azithromycin-treated animals showed a significant reduction in all clinical signs (p<0.05) compared to controls. Interleukin-6 (p<0.05), nuclear factor-kappa B protein expression (p<0.01), and MMP-2 activity (p<0.05) in conjunctival homogenates were significantly reduced compared with the control animals. MMP-2 gene expression showed a tendency to decrease in the azithromycin group (p=0.063). Mucus secretion by goblet cells and the macrophage count in conjunctival tissue were also decreased in the azithromycin group (p<0.05).

**Conclusions:**

These results suggest that azithromycin administration ameliorates induced inflammation effects in a rat model of acute conjunctivitis.

## Introduction

Azithromycin is a second-generation macrolide antibiotic that inhibits the synthesis of bacterial proteins. In ophthalmology, azithromycin is used topically in treating purulent bacterial conjunctivitis [1] and conjunctivitis caused by *Chlamydia trachomatis* [2,3]. Macrolide antibiotics are known for their efficacy in treating acute infections [4]. In addition to antibiotic activity, several studies have shown that azithromycin could play an anti-inflammatory role. The mechanisms of action for the anti-inflammatory properties of the macrolides are still being investigated, but they are multifactorial and unclear [5]. Anti-inflammatory activity has been evaluated in in vivo and in vitro studies that demonstrated azithromycin action on several proinflammatory factors. Evidence has accumulated over the last few years that part of the activity of macrolides is not mediated through their traditional antimicrobial effect [6].

Conjunctivitis is an inflammatory process of the conjunctiva and eyelids, and is usually associated with viral or bacterial infection [7]. Recently, there has been interest in the clinical application of azithromycin in treating infectious and inflammatory processes such as recurrent chronic multifocal osteomyelitis [5], asthma [8], cystic fibrosis [9], coronary artery disease [9], and concomitant ocular infection with *Chlamydia pneumonia* [10-12].

Early studies on the effect of azithromycin on human endothelial cells [13] demonstrated anti-inflammatory effects, independent of its antibiotic action, corroborated by studies demonstrating inhibition of proinflammatory factors [14]. Azithromycin also decreases transendothelial migration of neutrophils [15] and monocytes [16] involved in the genesis of inflammation [4,6,17]. Consequently, some authors suggested that azithromycin could have a potential role in treating infectious pathology with an inflammatory component, and suggested that this macrolide antibiotic might be the therapy of choice for infection-associated inflammation [17]. Although current evidence shows that azithromycin has beneficial effects in pathologies with an inflammatory component, a pathogen was also involved in almost all studies [18-21], with the exception of a murine model of corneal inflammation induced by thermal cautery [22]. Whether the beneficial effects were due to anti-inflammatory, antibiotic, or combined actions remains obscure.

The present study employs a model of lipopolysaccharide (LPS)-induced conjunctivitis in rats not associated with pathogens to investigate whether the anti-inflammatory properties might be demonstrated independently from any active microbial infection. LPS is an outer membrane component of Gram-negative bacteria recognized by the immune system as a pathogen-associated molecular pattern. LPS binds the Toll-like receptor 4/cluster of differentiation 14 complex on the surface of mammalian cells and activates cell signaling pathways to induce expression of inflammatory genes, including interleukin-1b, interleukin-6 (IL-6), and tumor necrosis factor-α (TNF-α). Peripheral injection of LPS in rodents stimulates cells of the innate immune system and increases the production of inflammatory cytokines [6,23].

The hypothesis to be tested is that azithromycin 15 mg/g eye drops (Azyter, Laboratoires Théa, Clermont-Ferrand, France) attenuate the inflammatory response to subconjunctivally injected LPS in a model of acute conjunctivitis in rats. In addition, inflammatory markers were quantified to assess genetic, protein, and morphological alterations.

## Methods

### Animals and study groups

This study was conducted in accordance with the National Institute of Health Animal Care and Use Committee protocols, the Association for Research in Vision and Ophthalmology Statement for the Use of Animals in Ophthalmic and Vision Research, as well as the guidelines of the Animal Ethics Committee of the University of Navarra. Animals were housed in a 12 h:12 h light-dark cycle and were provided food and water ad libitum. Fifty-four Wistar rats were divided in two groups; one group of 30 rats was set up to determine the maximum time point for inflammation, and the second group of 24 rats was used to determine the azithromycin anti-inflammatory effect. All animals were anesthetized with an intraperitoneal injection of ketamine (75 mg/kg, Imalgene 1000, Merial, France) and xylazine (10 mg/Kg, Rompun 2%, Carlier, Spain).

**Figure 1:**
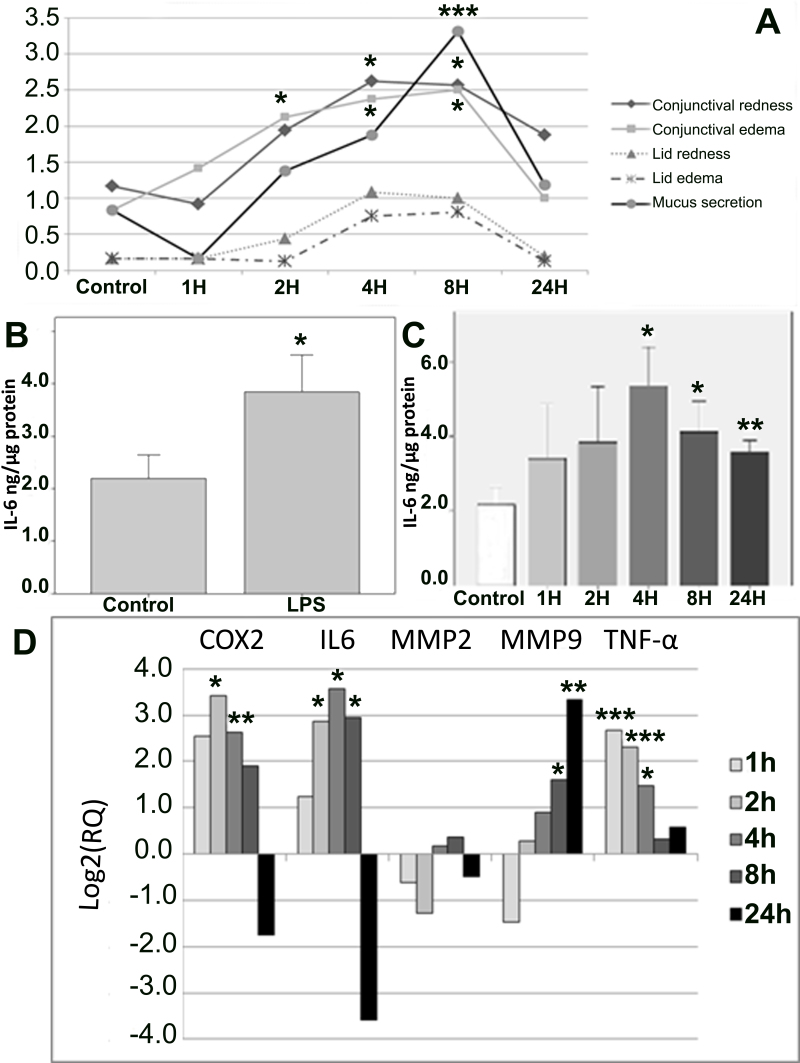
Results obtained at the maximum time point for inflammation. **A**: Clinical signs score obtained by three blind independent observers. **B** and **C**: Interleukin-6 (IL-6) levels determined in conjunctival tissue, control versus LPS-treated group and control vs. time-course lipopolysaccharide (LPS)-treated groups, respectively; data are expressed as mean±standard error of mean (SEM). **D**: Real time polymerase chain reaction (RT–PCR) expression analysis of cyclooxygenase-2 (*COX-2*), *IL-6, MMP-2, MMP-9*, and *TNF-α* proinflammation genes as a function of time in the LPS-treated group compared to the control group; data are expressed as log2 (relative quantity [RQ]). All data were analyzed with SPSS 15.0 software or DataAssist v2.0. (*p<0.05; **p<0.01, and ***p<0.001 versus control)

To determine the maximum inflammation time point, the animals received one subconjunctival injection in each eye of 30 µl of LPS (from *Escherichia coli* 055:B5 3 mg/ml; Sigma-Aldrich Química SA, Tres Cantos, Spain) or saline (NaCl 0.9%, B Braun España, Barcelona, Spain, control group, n=5) using a Hamilton high precision syringe (Gastight 1702LT; Hamilton Colorado, Reno, NV; Figure 1). Five animals were euthanized, under anesthesia, by cervical dislocation at 1, 2, 4, 8, and 24 h after injection (groups G1 to G5). Before the animals were euthanized, images of ocular inflammation were obtained (Topcon TRC 50FX camera; Topcon Corporation, Tokyo, Japan; Figure 1D), and clinical signs of inflammation (mucus secretion, conjunctival and lid edema and redness) as reported by other authors [7] were graded by three blind independent evaluators.

**Figure 2:**
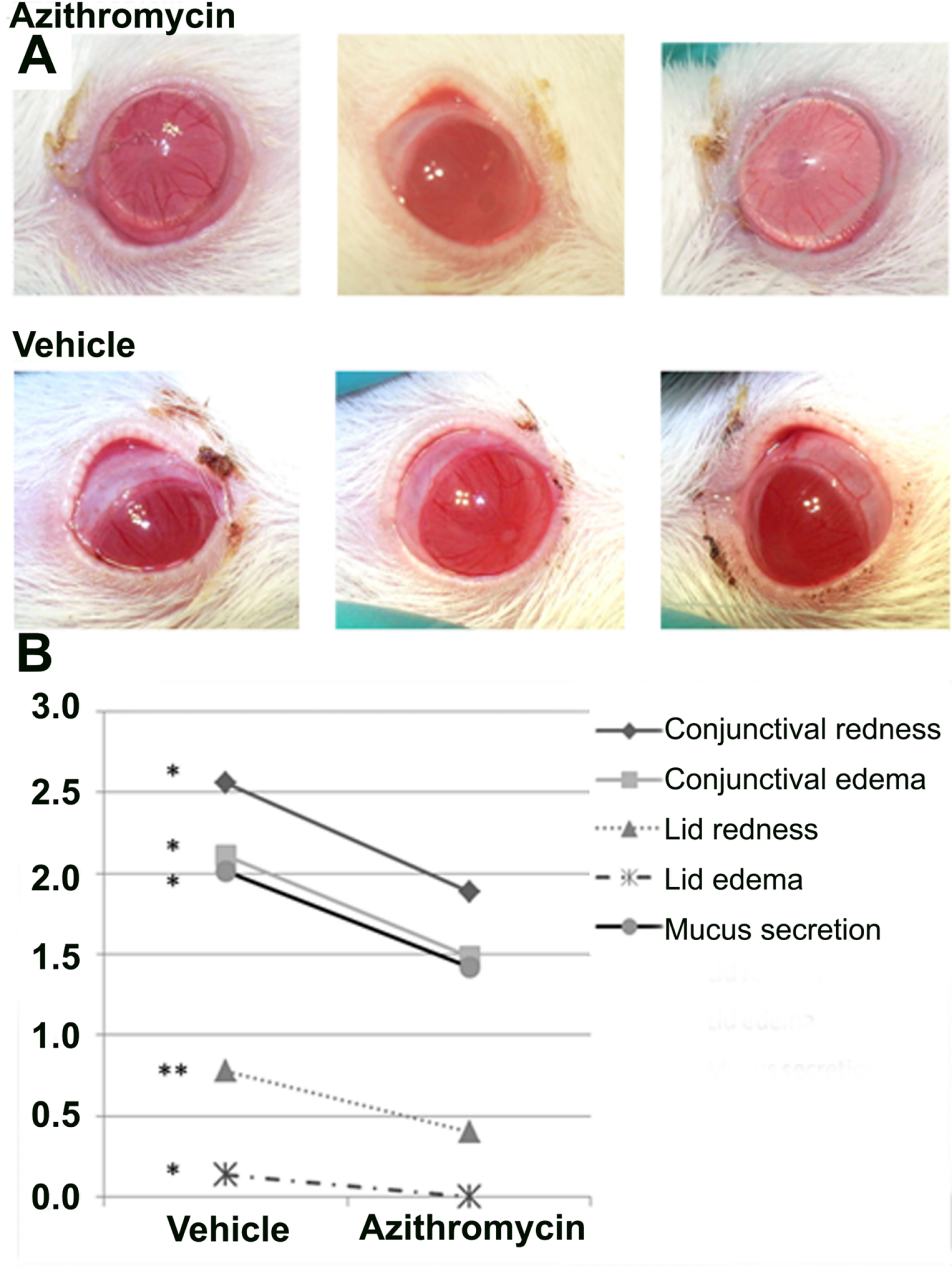
Anti-inflammatory effect of azithromycin in clinical signs. **A**: Pictures of azithromycin-treated eyes and vehicle-treated eyes obtained with Topcon TRC 50FX camera at 6 h post lipopolysaccharide (LPS) injection. **B**: Clinical sign scores were obtained by three blind independent observers. A minimum of ten animals per group were included. All data were analyzed with SPSS 15.0 software (*p<0.05 and **p<0.01 versus control)

To determine the anti-inflammatory effects of azithromycin, the rats were divided into two groups of 12 and received topical ocular azithromycin 15 mg/g or vehicle (Miglyol, Thea Laboratoires, Clermont-Ferrand, France). In total, six doses (25 µl) were administered in both eyes as one dose twice a day for three days before subconjunctival LPS injection (3 mg/ml). Azithromycin was administered according to the approved clinical regimen, i.e., twice a day for three days (i.e., a total of six drops) with the last drop 1 h before LPS injection. This results in much higher azithromycin concentration in the conjunctival tissue as after one drop there is little azithromycin in conjunctiva [24]. It is important to have the “real” clinical concentration to judge the impact of this regimen, and as the disease lasts only 24 h in this acute model, we had to instill azithromycin before LPS induction. If not, the inflammatory phenomenon ends before the loading concentration of azithromycin is obtained; thus, we could not observe an effect. Six hours post-injection, i.e., the time point for maximum inflammation, the animals were euthanized as for the previous groups (Figure 2). Conjunctival tissues were immediately frozen with dry ice for protein and RNA extraction.

### Quantitative real-time polymerase chain reaction

Total RNA was isolated from rat tissue using the ABI PRISM 6100 Nucleic Acid PrepStation (Applied biosystems, Life Technologies, Carlsbad, CA). Subsequently, the quantity and quality of purified mRNA were checked with a NanoDrop spectrophotometer (NanoDrop Technologies, Montchanin, DE) at 260/280. Using the qScript cDNA SuperMix kit (Quanta Biosciences Inc., Gaithersburg, MD), 1000 ng of each mRNA was reverse transcribed with the following conditions using a 2720 Thermal cycler: 5 min at 25 °C, 30 min at 42 °C, and 5 min at 85 °C (Applied Biosystems, Life technologies).

Predesigned and validated gene-specific TaqMan Gene Expression assays from Applied Biosystems, Life technologies were used for quantitative real-time polymerase chain reaction (RT–PCR). Every set contained gene-specific forward and reverse primers and fluorescence labeled probes. Probes span an exon junction and do not detect genomic DNA. The PCR reaction volume was 20 µl containing 1 µl cDNA, 10 µl TaqMan 2× Universal PCR Master Mix (Applied Biosystems, Life technologies), 1 µl predesigned and validated gene-specific TaqMan Gene Expression Assay 20× (Applied Biosystems, Life technologies), and 8 µl water. The ABI Prism 7300 real-time PCR system (Applied Biosystems, Life technologies) was used to amplify the five selected genes from the inflammation pathway, IL-6, matrix metalloproteinase-2 (MMP-2), MMP-9, TNF-α, and cyclooxygenase-2 (COX-2) in three parallel runs for each sample on a 96-well reaction plate with the following protocol: 2 min at 50 °C, 10 min at 95 °C, and 42 cycles of 15 s at 95 °C, 1 min annealing and extension at 60 °C. For further statistical evaluations, the level of applied housekeeping gene was used as the endogenous control for data normalization. Relative quantification studies were made from collected data (threshold cycle numbers, referred to as C_t_) with 7300 System SDS software 1.3 (Applied Biosystems, Life technologies). The relative quantity of the gene-specific mRNA was calculated with DataAssist v2.0.

### Extraction and determination of total protein concentration

The extracted conjunctival tissue was homogenized on 75 µl phosphobuffer (Sigma-Aldrich Química SA) using an Ultra-Turrax (IKA) and centrifuged at 13,000 rpm for 20 min at 4 °C. Supernatant was collected and protein content determined with Bradford’s method as previously described [25]. Bradford´s method is based on the binding of Coomassie Brilliant Blue Reagent (Bio-Rad, Temecula, CA), to a protein sample, and comparing this binding to a standard curve generated by the reaction of known amounts of a BSA standard protein (Sigma). The intensity of the color obtained is measured at 595 nm and 450 nm for a more sensitive determination.

### Western blotting for nuclear factor-kappa B

Equal amounts of conjunctival homogenates (3,5 µg) were subjected to western blot as previously described with some modifications [26]. Briefly, samples were mixed with Laemmli buffer (Bio-Rad, Hercules, CA) and boiled for 5 min. Samples were separated on 9% sodium dodecyl sulfate polyacrylamide gel electrophoresis (SDS-PAGE) gels and transferred to a nitrocellulose membrane. After blocking with 2% enhanced chemoluminescence Advance blocking agent (w/v; GE Healthcare, Fairfield, CT) and 0.1% Tween-20 (w/v) in tris buffered saline (TBS) for 1 h at room temperature, membranes were exposed to the primary antibody (1:500, antinuclear factor-kappa B [anti-NF-κB], Santa Cruz Biotechnology Inc., Santa Cruz, CA) overnight (at 4 °C) followed by incubation with a horseradish peroxidase-conjugated goat antirabbit antibody (sc2054; 1:5000, Santa Cruz Biotechnology Inc.). Signals were detected with an enhanced chemoluminescence kit (ECL-Advance western blotting detection kit, GE Healthcare) and signal captured with ImageQuant 400 (GE Healthcare). The relative intensities of the immunoreactive bands were analyzed by densitometry with ImageQuant TL software (GE Healthcare). The loading was verified with Ponceau S red, and the same blot was stripped and reblotted with an anti-β-actin monoclonal antibody (Sigma-Aldrich Química SA) to normalize the NF-κB levels. Each assay was repeated at least three times to ensure accuracy.

### Gelatin zymography assay for matrix metalloproteinase-2 activity

MMP-2 activity was quantified with gelatin zymography on subconjunctival tissue homogenates. Non-reducing sample buffer (62.5 mM Tris-HCl, pH 6.8; 10% glycerol; 0.1% bromophenol blue) was mixed with 8 µg of total protein from homogenate supernatants and electrophoresed directly on 9% SDS-polyacrylamide gels (SDS–PAGE) containing 0.1% gelatin (w/v). After electrophoresis, gels were washed 4 times for 20 min at room temperature in a 2.5% (v/v) Triton X-100 solution to remove excess SDS, transferred to a solution (Zymogram development buffer, Bio-Rad), and incubated for at least 18 h at 37 °C. Protein fixation was developed by incubating the gels for 15 min with 50% methanol/7% acetic acid and then washing for 30 min (six times, 5 min each) with distilled water. Then the gels were stained for 1 h with GelCode Blue Stain Reagent (Pierce, Rockford, IL), counter-stained with distilled water, and analyzed with ImageQuant TL software (GE Healthcare) after densitometric scanning of the gels. The active MMP-2/(active MMP-2+proMMP-2) intensity ratio was designated the MMP-2 activation ratio. Each zymography assay was repeated at least three times to ensure accuracy.

### Interleukin-6 determination with enzyme-linked immunosorbent assay

IL-6 levels were determined with enzyme-linked immunosorbent assay (ELISA; PeproTech, Rocky Hill, NJ, and Gen Probe, San Diego, CA, respectively) following manufacturers’ instructions. Equal quantities of total protein were used. Data are expressed on ng/µg of total protein for IL-6.

### Tissue processing for histological analysis

Immediately after euthanasia, conjunctival tissue was extracted and processed for histology. Briefly, tissue was fixed in Davidson’s solution (10% Glacial acetic acid, 30% ethanol [95%], and 20% formaldehyde [4%]) for 24 h and then in formaldehyde 4% for 24 h, followed by dehydration with a graded series of alcohol and paraffin embedded.

### Periodic acid-Schiff staining, conventional staining, and measurement of area of mucus secretion

Mucus secretion by goblet cells in the conjunctival epithelium was studied with periodic acid-Schiff (PAS), a specific technique for mucopolysaccharides. Four eyes of each group (azithromycin and vehicle treated) were fixed in Davidson’s solution as described above. Paraffin-embedded tissue was then subjected to microtomy, and 3 μm slides were obtained and exposed to conventional PAS staining to evaluate morphological changes. The mucin secretion stains purple, and the nuclei stains blue. Areas of mucopolysaccharides were measured by three independent observers in 40X photography by using Adobe Photoshop 7.0 lace and histogram tools in two PAS-stained samples of each animal. Areas data are presented as pixels (mean±standard error of the mean [SEM]) and are shown in Table 1.

**Table 1:** Parameters from all the evaluated experiments.

**Clinical signs score**										
Groups	**Conjuntival redness**	p	**Conjuntival edema**	p	**Lid redness**	p	**Lid Edema**	p	**Mucus secretion**	p
**Vehicle**	2.53±0.20		2.10±0.19		0.76±0.07		0.16±0.05		2.11±0.18	
**Azithromycin**	1.88±0.19	**<0.05**	1.48±0.14	**<0.05**	0.40±0.07	**<0.01**	0.00±0.00	**<0.05**	1.43±0.19	**<0.05**

**RNA expression**										
Groups	**COX-2 (RQ)**	p	**IL-6 (RQ)**	p	**MMP-2 (RQ)**	p	**MMP-9 (RQ)**	p	**TNF-α (RQ)**	p
**Vehicle**	1		1		1		1		1	
**Azithromycin**	1.745	ns	1.038	ns	0.433	0.061	0.605	ns	0.828	ns

**Protein analysis, macrophage infiltration and mucopolysacharide measurement**										
Groups	**IL-6 (ng/µl)**	p	**NF-κβ (%)**	p	**MMP-2 activity (%)**	p	**PAS staining (pixels)**	p	**CD 68 (cell number)**	p
**Vehicle**	2.03±0.45		100±3.4		100±6.2		429926±105314		134.5±29.6	
**Azithromycin**	0.70±0.10	**<0.05**	84.18±3.3	**<0.01**	80.7±3.9	**<0.05**	154685±38542	**<0.05**	27.8±9.9	**<0.05**

Data are represented as mean± Standard Error of Mean (SEM) *p<0.05 versus vehicle.

### Determination of inflammatory cell infiltration with immunohistochemistry and immunofluorescence

It is important to determine inflammatory cell infiltration into the damaged area; immunohistochemical analysis for cluster of differentiation (CD)4+ T cells as well as immunofluorescence for CD68 (specific marker for macrophages) localization was performed.

Paraffin-embedded conjunctival tissue sections were subjected to blocking of endogenous peroxidase activity. Macrophages were detected with indirect immunofluorescence. Antirat CD68 (AbDSerotec, Dusseldorf, Germany) was used as the primary antibody (1:100 diluted in phosphate buffered saline (PBS) without calcium and magnesium from Sigma-Aldrich, D8537, in goat normal serum 1:20) and goat antimouse Alexa Fluor 488 (Molecular Probes, Paisley, UK) as the second antibody (1:200 in PBS). Vectashield Mounting Media (Vector Laboratories, Peterborough, UK) with 4',6-diamidino-2-phenylindole (for nuclear staining) were used. Then samples were visualized under a fluorescence microscope with appropriate filters for correct visualization.

### Statistical analysis

Values are reported as mean±SEM. Statistical significance was evaluated with analysis of variance (ANOVA) or Kruskal–Wallis test to determine the maximum time point for inflammation and the *Student* *t* test or the Mann–Whitney U-test to assess the anti-inflammatory effect of azithromycin. After a significant ANOVA or Kruskal–Wallis test, comparisons between groups were made with the Bonferroni post-hoc or Mann–Whitney U-test, respectively. Statistical significance was accepted at the 95% confidence level (p<0.05), and analysis was performed by using the computer program SPSS (v. 15.0, SPSS Inc. Chicago, IL). DataAssist v 2.0 (Applied Biosystems) was used for gene expression quantitative analysis. Histological analyses for grading, cell counting, and area measurement were performed by three blind independent investigators, and the results obtained are shown in Table 1 (mean±SEM). All data are expressed as the treatment means±SEM.

## Results

### Maximum time point for inflammation

#### Clinical signs

To establish the time point for maximum inflammation, clinical signs and IL-6 levels were evaluated. The analysis of clinical sign scores (Figure 1A) obtained from independent observers showed significant differences in the ANOVA test between the control group and groups G1–G5 for conjunctival redness, conjunctival edema, and mucus secretion (p=0.049, p=0.039, and p=0.0001, respectively). Comparisons of each group to the control group showed a peak of inflammation in groups G3 and G4 (i.e., 4 and 8 h after LPS injection), with significant differences in conjunctival redness (p=0.024 and p=0.030, respectively), conjunctival edema (p=0.020 and p=0.013, respectively), and mucus secretion (p=0.069 and p<0.001, respectively).

#### Interleukin-6 determination with enzyme-linked immunosorbent assay

IL-6 levels were almost doubled by LPS (p=0.033) as averaged from pooled individual values of all G1 to G5 animals (Figure 1B). Moreover, IL-6 levels were significantly higher in animals euthanized 4, 8, and 24 h (p=0.014, p=0.024 and p=0.004, respectively) after LPS injection (Figure 1C).

#### Quantitative real-time polymerase chain reaction

The quantitative real time polymerase chain reaction expression analysis of inflammation-involved genes showed significant differences in groups G2, G3, and G4 in several genes compared to the control group (Figure 1D). The *TNF-α* gene, one of the principal genes involved in activating the inflammation pathway showed a significant increase at early time points (G1, p=9×10^−4^; G2, p=6×10^−4^; G3, p=0.014). Similarly, the expression of *COX-2* and *IL-6* genes underwent a significant increase at intermediate time points G2 (p=0.049 and p=0.042, respectively), G3 (p=0.005 and p=0.017, respectively), and G4 (p=0.019, only in *IL-6*). Meanwhile, the analysis of the *MMP-9* gene showed significant activation only at late time points (G4, p=0.011; G5, p=0.001). *MMP-2* gene expression did not reach statistical significance.

Since most of the genes and protein levels were significantly higher between 4 and 8 h, 6 h was selected as the point of maximum inflammation and used for the subsequent evaluation of the azithromycin anti-inflammatory effect.

### Anti-inflammatory effect of azithromycin

#### Clinical signs

The analysis of clinical sign scores comparing the azithromycin-treated group with the vehicle-treated group (Figure 2A,B) showed a significant decrease in all parameters: redness and edema, both conjunctiva and lid (p=0.034; p=0.024; p=0.023 and p=0.003, respectively), and the mucus secretion score (p=0.015).

#### Interleukin-6 determination with enzyme-linked immunosorbent assay

**Figure 3:**
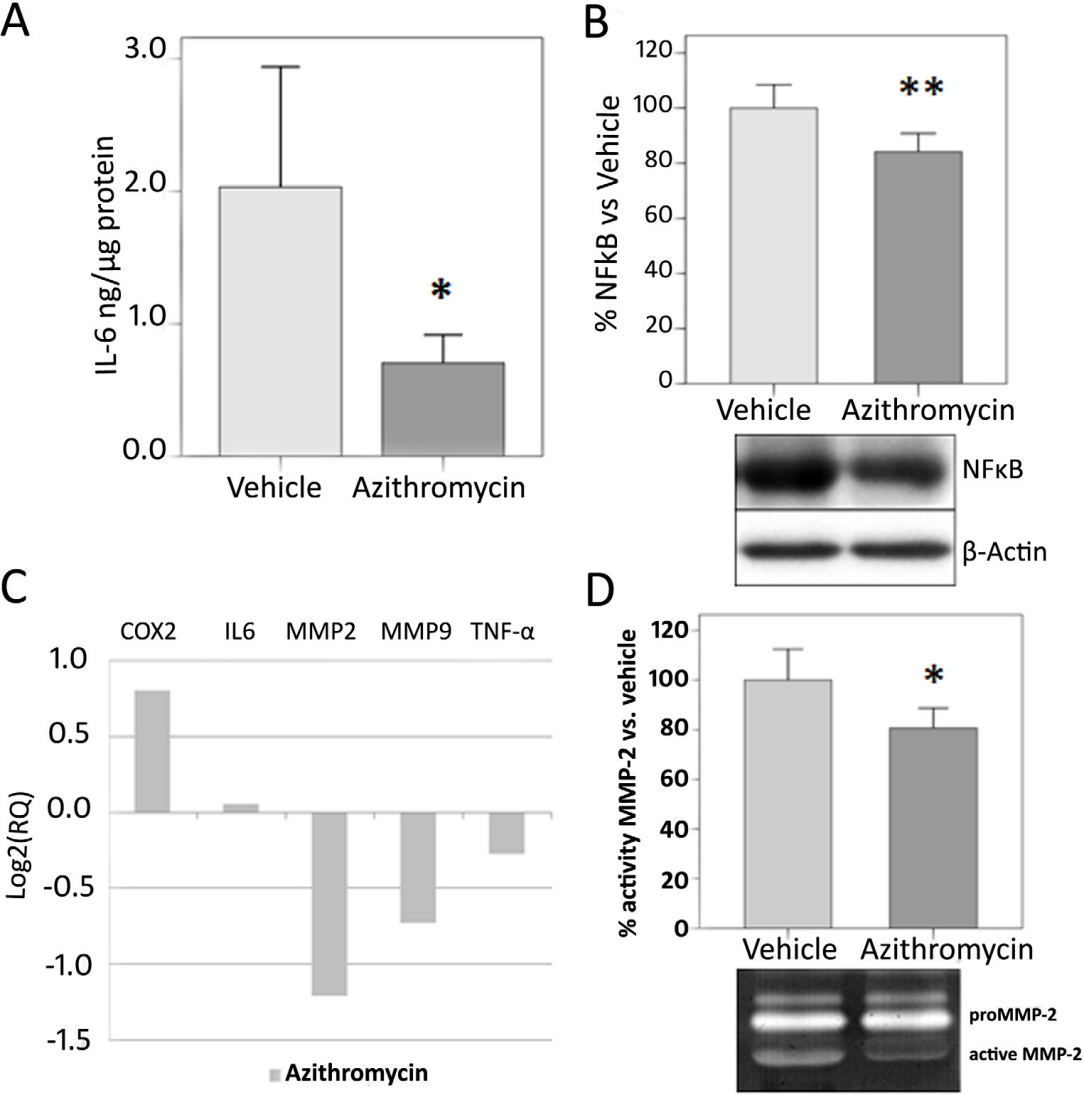
Biochemical analysis of azithromycin treatment group versus control group. **A**: Interleukin-6 (IL-6) levels are expressed as ng/µg of total protein in conjunctival tissue. Azithromycin treatment reduced IL-6 levels in conjunctival tissue. **B**: Measurement of nuclear factor-kappa B (NF-κB) expression in conjunctival tissue. Bars represent mean±SEM. β-actin was blotted as an internal control. **C**: Real time polymerase chain reaction (RT–PCR) expression analysis of cyclooxygenase-2 (*COX-2*), *IL-6, MMP-2, MMP-9*, and *TNF-α* proinflammation genes in the azithromycin-treated group. Change is relative to the vehicle-treated group (reference). Data are expressed as log2(RQ). **D**: Representative zymogram showing MMP-2 activity and the results shown as percentage of the azithromycin-treated group versus the vehicle-treated group. Bars represent mean±SEM. A minimum of ten animals per group were included. All data were analyzed with SPSS 15.0 software or DataAssist v2.0. (*p<0.05 versus vehicle).

To determine IL-6 levels as a marker of inflammation, ELISAs were performed on conjunctival tissue homogenates (Figure 3A). The azithromycin group showed a statistically significant reduction in IL-6 levels (p=0.043) compared to the vehicle group.

#### Western blot analysis of nuclear factor-kappa B

Western blot for NF-κB in conjunctival homogenates revealed significantly less NF-κB (p=0.009) expression in the azithromycin than in the vehicle group (Figure 3B).

#### Quantitative real-time polymerase chain reaction

Regarding RT–PCR gene expression analysis, there were no significant differences in any of the studied genes between the azithromycin-treated group and the vehicle-treated group (Figure 3C). However, the *MMP-2* and *MMP-9* genes showed nearly significant reduction in expression in the azithromycin-treated group (p=0.061 and p=0.100, respectively).

#### Gelatin zymography of matrix metalloproteinase-2 activity

Figure 3D shows zymographs for MMP activity that demonstrate a statistically significant reduction (p=0.018) in MMP-2 activity in the animals treated with azithromycin compared with the vehicle-treated animals.

### Morphological evaluation of tissue sections and mucus secretion assessed with periodic acid-Schiff staining

After the tissue sections were processed for histology, images were analyzed for correlation of biochemical changes with morphologic alterations. Results are summarized in Table 1.

**Figure 4:**
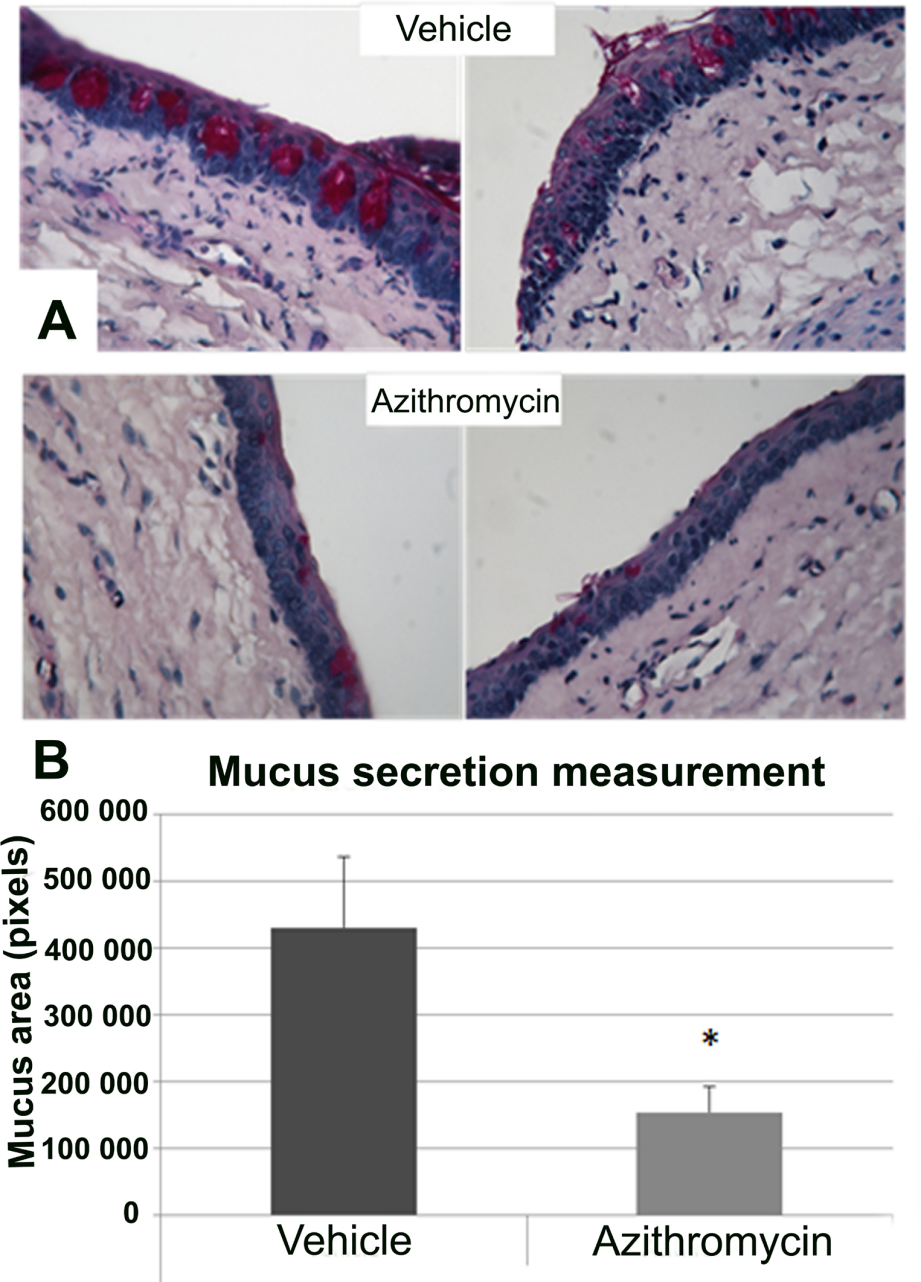
Mucus secretion analysis in conjunctival tissue. **A**: Periodic acid-Schiff (PAS) staining in slides of vehicle (upper) and azithromycin (lower). Azithromycin showed a reduction in mucin secretion (purple) compared with vehicle-treated animals. Magnification: 40X. **B**: Results obtained for measurements performed in PAS-stained sections. Data are represented as mean±standard error of mean (SEM). Four eyes per group were included. All data were analyzed with SPSS 15.0 software. (*p<0.05 versus vehicle)

Figure 4A shows PAS staining in conjunctival sections demonstrating a clear reduction in mucopolysaccharide production by goblet cells in the azithromycin- vs. vehicle-treated animals. Moreover, the area of mucus in the vehicle-treated animals was significantly larger (p<0.05) than in the azithromycin-treated group, as shown in Figure 4B.

### Determination of inflammatory cells infiltration with immunohistochemistry and immunofluorescence

**Figure 5:**
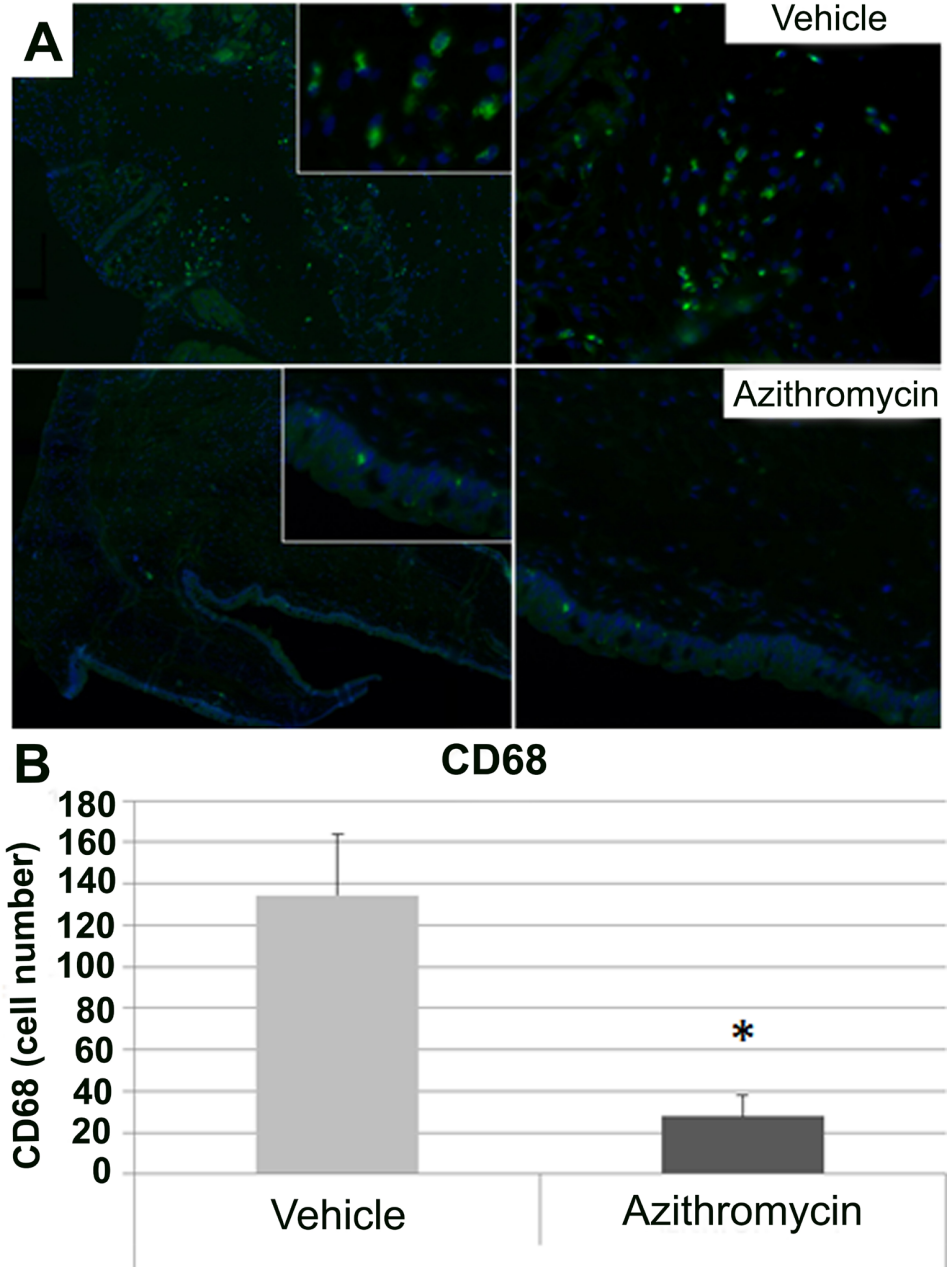
Macrophage infiltration analyzed in conjunctival tissue. **A**: Cluster of differentiation 68 (CD68) positive cells in slides of vehicle (upper; magnification: left 5X, right: 40X) and azithromycin (lower; magnification: left 5X, right: 40X). The azithromycin-treated group showed a decrease in the number of macrophages compared with the vehicle-treated animals. CD68 (macrophages): green; 4',6-diamidino-2-phenylindole (nuclei): blue. **B**: Results obtained for counting of CD68 positive cells in immunofluorescence slides. Data are represented as mean±standard error of mean (SEM). Four eyes per group were included. All data were analyzed with SPSS 15.0 software. (*p<0.05 versus vehicle).

Immunofluorescence for CD68, a marker of macrophage infiltration, showed a statistically significant (p<0.05) reduction of the number of CD68 positive cells in the animals treated with azithromycin versus the control animals (Figure 5A,B).

## Discussion

The present study is the first to demonstrate the anti-inflammatory effect of azithromycin in a rat model of acute conjunctivitis induced by LPS. A reduction in inflammatory clinical signs, gene and protein expression of several proinflammatory molecules (such as IL-6, MMP-2 activity, and NF-κB), and macrophage infiltration in damaged tissue as well as a reduction in conjunctival mucus area were observed.

In addition to antibiotic activity, azithromycin also possesses anti-inflammatory activity [27], though relatively weak compared to glucocorticoids. However, azithromycin can achieve much higher tissue concentrations, has a long tissue half-life at clinical doses, and has fewer adverse effects than corticoids [28,29]. Consequently, this antibiotic may have utility for certain inflammatory ocular surface diseases [29].

Pharmacokinetic studies have confirmed the availability of azithromycin in tears, conjunctiva, and cornea 24 h after a single instillation and twice-daily instillation for three days of 1.50% azithromycin in the rabbit eye [24]. Such findings permit the hypothesis that the effect observed in the conjunctiva is caused by azithromycin. The clinical signs data obtained in the present study demonstrate that azithromycin was able to significantly reduce inflammatory signs following LPS injection. Lid and conjunctival redness and edema and mucus secretion were less severe in animals treated with azithromycin compared with those given vehicle, suggesting that azithromycin as well as other macrolides [30] are able to prevent clinical alteration signs of inflammation as observed in experimental models of corneal refractive surgery [11], uveitis in rabbits [31] and humans [4,17,21,23].

These clinical signs results agree with those obtained for protein expression; IL-6 levels were higher in animals with LPS compared with the control animals, and azithromycin reduced IL-6 levels after topical administration for three days following LPS. We have also demonstrated that azithromycin inhibits LPS-induced NF-κB activation in this model, suggesting an anti-inflammatory effect of topical azithromycin. Macrolides inhibit the production of many proinflammatory cytokines, such as IL-1, IL-6, IL-8, and TNF-α, perhaps by suppressing the transcription factor NF-κB or activator protein-1 [5]. MMP-2 activity is reduced after topical administration of azithromycin compared to controls, suggesting that azithromycin has an important inhibitory role in synthesizing and degrading extracellular matrix. Proteolysis could be increased by LPS with subsequent tissue damage. After azithromycin, degradation of extracellular matrix could diminish as observed in vitro in fibroblasts [32]. Furthermore, researchers have demonstrated that azithromycin suppresses expression of matrix metalloproteinases in human corneal epithelial cells stimulated by zymosan [33]. Moreover, in vivo studies using carrageenan and in vitro murine macrophages stimulated with LPS show a reduction in the generation of some mediators and cytokines involved in the inflammatory process, such as arachidonic acid metabolites, nitric oxide, TNF-α , interleukin-1b, and IL-6 [23].

Gene expression analysis showed no significant differences between the azithromycin-treated animals and the vehicle-treated animals. However, MMP-2 and MMP-9 expression was almost significantly reduced by azithromycin. However, mRNA expression is first induced and lasts for a short period, and protein expression is subsequently induced. This means that effects at the gene expression level in this rat model are more difficult to demonstrate than those at the protein level.

Mucin production has been widely related to inflammatory pathologies such as asthma and bronchitis, suggesting an important role of these proteins in the establishment and progression of disease. Goblet cells in the normal conjunctiva are empty of mucus and secrete mucins to protect the eye. Moreover, chronic inflammatory diseases such as allergic conjunctivitis and early dry eye lead to increased goblet cells’ mucin secretion into tears and ocular surface [6,34,35]. The clinical signs (mainly mucus production) in the present study prompted determination of mucopolysaccharides production by goblet cells in conjunctival tissue slides with PAS staining. However, researchers have demonstrated that in inflammatory pathologies, e.g., bronchiectasis, there is excessive mucus secretion by goblet cells. The present study showed an increase in mucus production in the conjunctival tissue of the control animals versus the animals treated with azithromycin, suggesting that azithromycin seems to play an important role in reducing mucus secretion by conjunctival goblet cells [35].

Another important finding of this study is that azithromycin reduced the infiltration of inflammatory cells (macrophages) into areas of damage. An important aspect of inflammation is extravasation of neutrophils and inflammatory cells into the tissues; macrolides block formation of the adhesion molecules necessary for cell migration [5]. Macrophages are versatile cells intimately involved in all aspects of the immune response and the complex process of inflammation [16]. The decrease in the number of macrophages in this study agrees with observations at the gene and protein levels, as well as with clinical signs graded in a previous study. The study demonstrates that azithromycin decreases production of mucus by epithelial cells and biosynthesis of proinflammatory cytokines from macrophages and epithelial cells by inhibiting NF-κB [6]. Moreover, other authors have shown that inhibition of LPS-induced pulmonary neutrophilia and IL-1β concentrations in the lung are mediated through effects on alveolar macrophages [36] and inhibition of the in vitro production of IL-6, IL-8, and macrophage inflammatory protein-2 by human monocytes [6].

In conclusion, we have examined the effects of azithromycin on the LPS-induced acute conjunctivitis model in rats, and we found evidence that topical azithromycin inhibits the expression of inflammatory cytokines such as IL-6, MMP-2 activity, and NF-κB. Moreover, topical administration of azithromycin reduces the effects of acute inflammation such as mucus secretion and macrophage infiltration. Azithromycin could be a good alternative to corticoids to solve inflammation-derived alterations. Macrolides, far beyond their traditional antimicrobial properties, possess considerable anti-inflammatory properties, corroborated in the present study in an acute model of conjunctivitis. These results suggest that azithromycin is a promising agent for treating ocular surface inflammation in its clinical indications of non-bacterial conjunctivitis.
